# Antimicrobial resistance: Strengthening surveillance for public health action

**DOI:** 10.1371/journal.pmed.1004265

**Published:** 2023-07-12

**Authors:** Silvia Bertagnolio, Amitabh Bipin Suthar, Olga Tosas, Kitty Van Weezenbeek

**Affiliations:** 1 Surveillance, Prevention & Control, Division of Antimicrobial Resistance, World Health Organization, Geneva, Switzerland; 2 Atlanta, Georgia, United States

Antimicrobial resistance (AMR) is among the top ten global public health threats, demanding immediate attention and action [1]. To effectively tackle AMR, it is crucial to have a comprehensive understanding of the extent of the problem. Specifically, reliable, accurate, and representative information on the incidence of resistant infections is vital for monitoring the national and global scale of AMR, identifying emerging threats, and evaluating the impact of interventions. As part of *PLoS Medicine’s* Special Issue dedicated to AMR, Balasubramanian and colleagues shed light on the global magnitude of hospital-associated infections due to hospital acquired resistant infections (HARI) [2].

One of the strengths of the study is the combination of data from hospital-based point prevalence surveys (PPS) with estimates of hospitalization rates, resistance prevalence, and country population size to predict the number of HARIs globally, across country income categories, and within nations. Using this approach, the authors estimated approximately 136 million HARI annually worldwide between 2010–2020. To our knowledge, these are among the first global incidence estimates of HARI. However, as acknowledged by the authors, results heavily rely on the availability of PPS data, which is limited in some regions and countries. According to the authors, only 11 low- and middle-income countries report this information, often based on small sample sizes, which may introduce bias and limit the generalizability of their findings. Additionally, the study relies on extrapolating country-level infection rates and resistance prevalence from available but scarce data at the hospital level, thus introducing uncertainties and assumptions that could impact the accuracy of the results. The heterogeneity of data from various sources and countries, with differences in data collection methods, diagnostic criteria, and reporting practices, could also affect the reliability of the estimates. Furthermore, due to the lack of available data, the study reports infections in an aggregated manner, without distinguishing specific infectious syndromes, which hampers the identification of more targeted syndrome-based interventions.

Recent AMR burden estimates indicate that in 2019, 1.27 million deaths were attributed to drug resistant bacterial infections [3]. Comparing estimates from different sources is limited by a lack of standardized reporting. Balasubramanian et al. included six bacteria and 13 antibiotic classes, across all infectious syndromes. [2]. WHO’s Global Antimicrobial Resistance and Use Surveillance System (GLASS) currently recommends 11 antibiotic classes, eight bacteria (*Acinetobacter spp*., *E*. *coli*, *K*. *pneumoniae*, *Salmonella spp*., *S*. *aureus*, *Streptococcus pneumoniae*, *Shigella spp*., and *N*. *gonorrhoeae*) and blood, urine, feces, and urethral and cervical swabs as priority specimen types for routine AMR surveillance and reporting to WHO [4]. These criteria will be expanded and updated in the forthcoming WHO GLASS 2.0 manual. The Global Burden of Disease (GBD) estimates included 12 infectious syndromes, 18 antibiotic classes, and 23 bacteria [3]. A complete understanding of the AMR infection burden requires both hospital and community-based data. Targeted surveillance of community-acquired resistant infections can be challenging, generating results biased toward hospital-acquired infections. Only GBD estimated the burden of AMR in communities [3], although their estimates do not stratify AMR burden according to hospital and community settings.

The estimates from Balasubramanian et al. [2] further our understanding of the global magnitude of AMR in hospital settings, while highlighting the unreliability of existing AMR surveillance data. Currently, available estimates rely on fragmented and limited data, often derived from studies in high-income countries using differing methods, thus making regional or global consolidation extremely challenging. Standardizing the core infectious syndromes, pathogens, and antibiotic classes included in AMR surveillance would improve comparability between countries and eventually at a global level while allowing for supplemental local adaptation. To address this issue, and to accelerate the availability of representative data that can provide reliable global and national estimates of AMR over time, WHO proposes a two-pronged approach: (1) generating nationally representative data through strengthening and standardizing routine *passive* surveillance of AMR in clinical samples from patients with suspected infections and (2) *actively* conducting periodic nationally representative surveys to measure the prevalence and trends of AMR [5].

**Fig 1:**
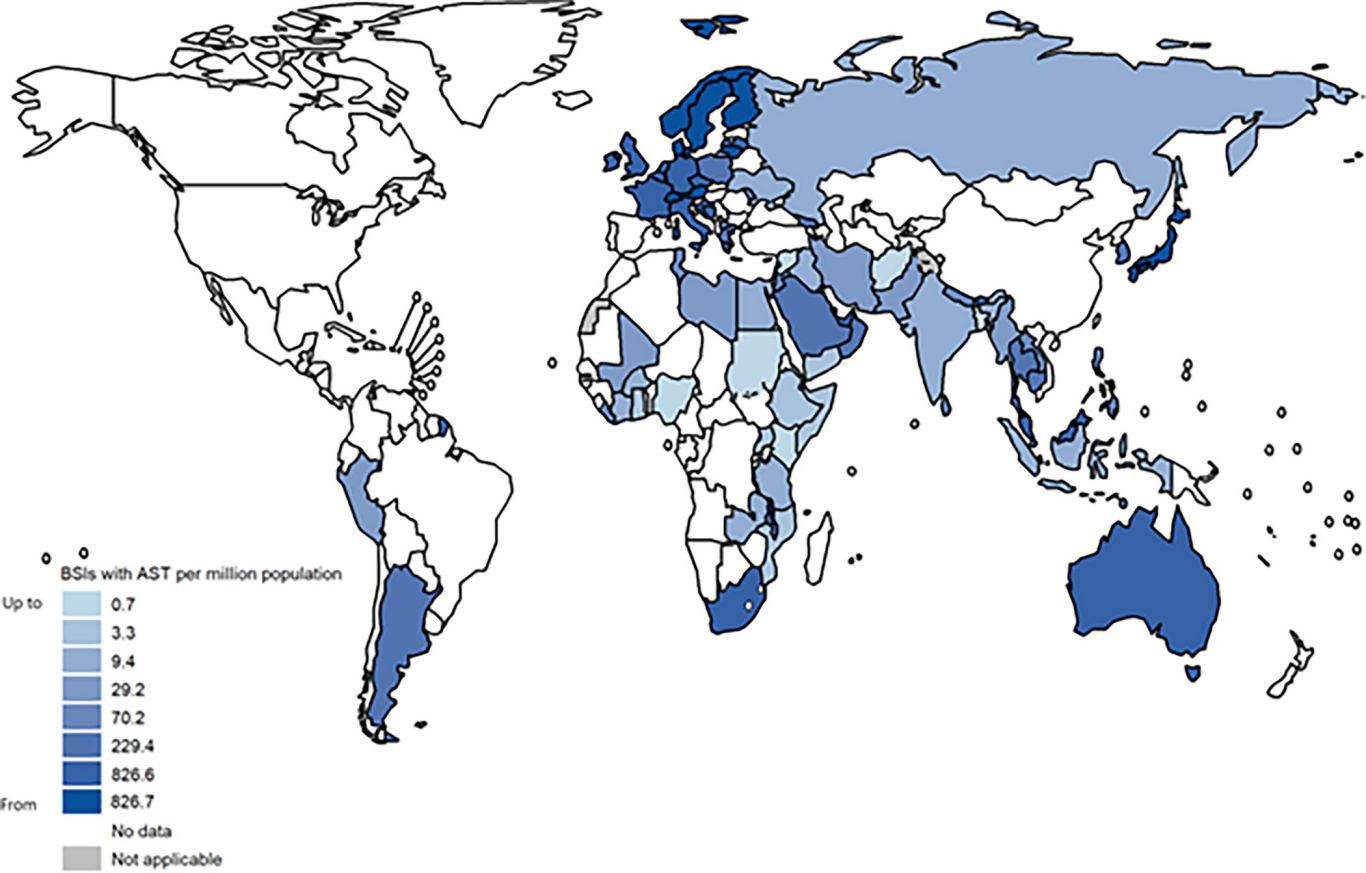
Bacteriologically confirmed bloodstream infections (BSIs) with antimicrobial susceptibility test (AST) results per one million population, reported to WHO GLASS in 2020 (adapted from [8]). In 2020, GLASS collated bacteriologically confirmed bloodstream infections due to the following pathogens: *Acinetobacter spp*., *Escherichia coli*, *Klebsiella pneumoniae*, *Salmonella spp*., *Staphylococcus aureus and Streptococcus pneumoniae*. Reporting shows gross heterogeneity in patient population testing coverage (relative to the total population in each country) for one of the most life-threatening infectious syndromes. Of note, several counties were not able to report 2020 AMR data to GLASS during the COVID-19 pandemic. (*The whomap R package used for map layers can be found at https://github.com/glaziou/whomap*).

Continuous AMR surveillance models are typically passive necessitating regular reporting of event data by all facilities that consult patients or test specimens. Routine and continuous surveillance systems for AMR improve access to timely and appropriate treatment, rapid detection of outbreaks and real-time monitoring of trends and effectiveness of interventions. However, where capacity for high-quality and high-(national) coverage is lacking, continuous surveillance may come with significant biases. Reporting to WHO GLASS shows that coverage of adequate microbiological diagnostic services varies greatly between countries and is often scarce in resource-limited settings (Fig 1). Other challenges include financial hurdles for patients resulting in limited uptake of testing and inaccurate and/or incomplete clinical data, often related to inadequate laboratory management systems. Furthermore, the quality assurance of routine laboratory testing is often not guaranteed through internal and external mechanisms. Learning from TB, in most low and middle-income countries it may take years to achieve sufficient coverage and quality of data for policy development in the absence of the implementation of other complementary methods, such as nationally representative surveys [6, 7].

Active AMR surveillance through periodic national AMR prevalence surveys offers a reliable, direct measurement of the prevalence of AMR for countries lacking high-quality and high-coverage surveillance systems. This is achieved through inclusion of a subset of surveillance sites selected using statistically meaningful probability sampling methods, to achieve national representativeness and data accuracy. Surveys are typically implemented following key scientific quality standards and standardized methods which ensure that the resultant data will be comparable within and between countries and over time. These surveys use the same active systematic syndrome-based approach for case finding and enhance national technical capacity, leading to improved patient management and development of robust continuous surveillance systems in the longer term. Alternative AMR surveillance options for countries that do not yet have continuous AMR surveillance systems of high-quality and high coverage, include the establishment of a sentinel system or lot quality assurance sampling. However, unlike the periodic nationally representative surveys, these approaches do not accurately measure the magnitude and trends of AMR in a country population and consequently do not fulfil the objectives of national and global monitoring of AMR. Finally, mathematical modelling can accelerate the availability of evidence on a global scale, but its value is contingent upon the quality and representativeness of the data used and the validation of its estimates.

It is crucial to consider service coverage or the attrition in the “cascade” from diagnosis to cure, when interpreting AMR surveillance and evaluating the impact of AMR. WHO guidance on the methodological principles of nationally representative AMR prevalence surveys considers the patient pathway within the healthcare setting when measuring the prevalence of AMR [9], focusing initially on bacterial bloodstream infections among individuals in need of acute hospital inpatient care, with the plan to expand to other infectious syndromes [9]. The nationally representative survey platform thus offers an opportunity for meaningful country-wide assessment of the estimated number of incident infections, the number of infections diagnosed, the proportion of diagnosed infections that receive treatment, and the proportion of treated infections that are successfully cured. By examining these indicators, we can gain insights into how access to healthcare services influences the magnitude of AMR over time, enabling targeted attention and intervention. AMR prevalence surveys can be nested with a WHO standardized approach to generate robust estimates on the mortality attributable to antimicrobial resistant bloodstream infections [4].

While much work remains to be done to implement reliable and timely AMR surveillance systems needed to control this silent pandemic, the estimates being generated by Balasubramanian et al., and other investigators, are a valuable starting point. These data help communicate the urgency and gravity of the problem and hopefully motivate countries to implement robust surveillance systems for generating reliable data to inform public health action.
